# Understanding socio-economic inequalities in the prevalence of asthma in India: an evidence from national sample survey 2017–18

**DOI:** 10.1186/s12890-021-01742-w

**Published:** 2021-11-15

**Authors:** Rashmi Rashmi, Pradeep Kumar, Shobhit Srivastava, T. Muhammad

**Affiliations:** 1 International Institute for Population Sciences, Mumbai, India; 2 Department of Survey Research & Data Analytics, International Institute for Population Sciences, Mumbai, India; 3 Department of Population Policies and Programmes, International Institute for Population Sciences, Mumbai, India

**Keywords:** Asthma; Socio-economic inequality; Decomposition; India

## Abstract

**Background:**

Today, over 300 million people reside with asthma worldwide and India alone is home for 6% of children and 2% of adults suffering from this chronic disease. A common notion of disparity persists in terms of health outcomes across the poor and better-off section of the society. Thus, there is a need to explore socio-economic inequality in the contribution of various factors associated with asthma prevalence in India.

**Methods:**

Data for the study were carved out from the 75th round of National Sample Survey (NSS), collected by the National Sample Survey Organization (NSSO) during 2017–18. The sample size for this study was 555,289 individuals, for which data was used for the analysis. Descriptive statistics were used to show the distribution of the study population. Further, bivariate and multivariate analysis was performed to identify the factors associated with Asthma prevalence. The concentration index was used to measure the inequality. Further, we used decomposition analysis to find the contribution of factors responsible for socio-economic status-related inequality in asthma prevalence.

**Results:**

The prevalence of asthma was 2 per 1000 in the whole population; however, the prevalence differs by age groups in a significant manner. Age, sex, educational status, place of residence, cooking fuel, source of drinking water, household size and garbage disposal facility were significantly associated with asthma prevalence in India. It was found that asthma was more concentrated among individuals from higher socioeconomic status (concentration index: 0.15; *p* < 0.05). While exploring socio-economic inequality for asthma, richest wealth status (53.9%) was the most significant contributor in explaining the majority of the inequality followed by the urban place of residence (37.9%) and individual from age group 45–65 years (33.3%). Additionally, individual aged 65 years and above (27.9%) and household size less than four members (14.7%) contributed in explaining socio-economic inequality for asthma.

**Conclusion:**

Due to the heterogeneous nature of asthma, associations between different socio-economic indicators and asthma can be complex and may point in different directions. Hence, considering the concentration of asthma prevalence in vulnerable populations and its long-term effect on general health, a comprehensive programme to tackle chronic respiratory diseases and asthma, in particular, is urgently needed.

## Background

With the passage of time and changing lifestyles, the world is combating the growing threat of non-communicable diseases (NCDs). According to the World Health Organization (WHO), NCDs are responsible for 71% of all deaths worldwide [1] and adds substantial health and economic burden to nations that are already battling communicable and infectious diseases. In India, Global Burden of Disease (GBD) Collaborators showed a state-level variation in epidemiological transition and found that the burden of NCDs like cardiovascular diseases, respiratory diseases and diabetes had escalated at an alarming rate [2]. While the contribution of cardiovascular diseases in total mortality of India was found to be the largest [3], the prevalence of respiratory disease named asthma had also increased by 8.6% during 1990–2016 [4]. According to the definition of WHO, Asthma is a chronic or long term condition that inflames and narrows the airways in the lungs from time to time causing chest tightness, shortness of breath, wheezing and coughing [5]. Today, over 300 million people reside with asthma worldwide [6] and India alone is home for 6% of children and 2% of adults suffering from this chronic disease [7].

Although Asthma contributes a smaller burden of total mortality among non-communicable diseases, it still poses a serious concern as most of the deaths caused are preventable [8]. With no exact cure, this disease can be triggered through genetic and environmental factors. An Australian cross-sectional study found that asthma among children was highly associated with the direct and indirect effect of genetics, environment and allergens [9]. A longitudinal cohort study from Tucson provides evidence that chronic asthma among adults was highly associated with their onset from childhood and persistent wheezing in early life [10]. Past research had also indicated that early onset of asthma was linked with age [11], sex [12], genetic factors [13], parental smoking [14], active smoking in childhood [15], preterm birth [16], larger families [17] and childhood obesity [18]. Besides these factors, few psychological determinants were significantly linked with asthma among individuals [19].

Extant research from India has shown the prevalence, trend, socio-economic, demographic and environmental predictors of asthma morbidity across different sections of the population [20–23]. A study using the second round of India Human Development Survey data linked the burden of asthma with households using unclean fuels, individuals who are lesser educated and those who belong to a poorer section of society [24]. Further, a study had shown the role of various occupations among adults in building the risk of four non-communicable diseases including asthma [23]. Studies had also linked the influence of stressful psychosocial circumstances and spatial heterogeneity with the asthma prevalence in India [25, 26].

Across developed and developing countries, the trend in asthma mortality has decreased with a steady increase in the prevalence in the past few years and the reasons for such increase are yet not defined [27]. Despite advancements in technology to diagnose and manage asthma in developed countries, a study from New York city reveals that poor housing condition, outdoor air pollution and noxious land uses can contribute higher incidence of asthma in urban neighbourhoods [28]. A study, further, revealed that adolescents residing in peri-urban areas of developing countries are more prone to asthma [29]. The same study shows that the history of cigarette smoking and indoor pollution increases the likelihood of reported and symptoms of asthma. This brings our attention to the situation of developing nations, which are already succumbed from infectious diseases and are continuously burdened with the people who are yet not diagnosed or are unaware with the risk they are carrying. The risk is, further, increased in a country like India where the burden of non-communicable diseases is escalating [30] along with a sharp rise in urban settlements of poor [31].

**Fig. 1:**
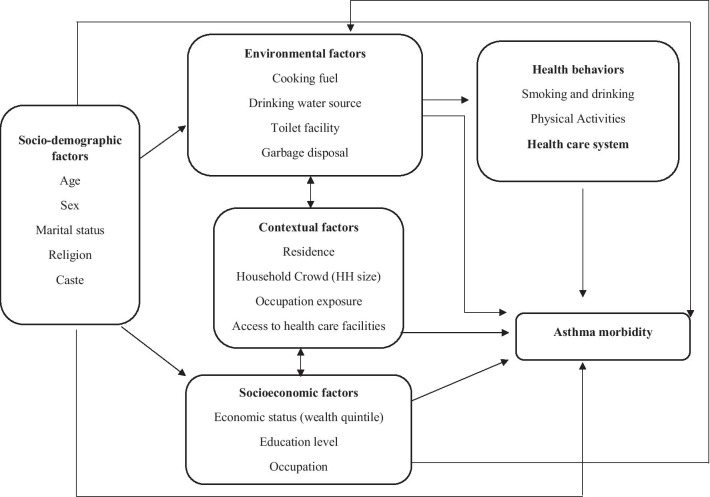
Conceptual framework of asthma morbidity

The rationale for the current analysis is as follows. First, despite having a minimal mortality, asthma remains to have constant threat due to no exact cure procedure. Moreover, the growing urbanization and industrialization and changing lifestyles have increased the chances of asthma prevalence in both poor and richer section of society. Second, so far minimal evidence from India had examined the extent of socioeconomic inequality in NCD prevalence especially asthma which remains to be highly dominating in both younger and older age groups of society [32]. Lastly, due to variations in geographical, environmental, social, economic and cultural factors across the states of India, a state-wise inequality in asthma prevalence among different socio-economic groups can present a reliable estimate across India. Therefore, as per the conceptual framework provided in Fig. 1, the current study aims to explore the factors associated with asthma and the contribution of those factors in socioeconomic inequality in the prevalence of asthma in India.

## Data and methods

### Data source

Data for this study were carved out from the 75th round of National Sample Survey (NSS), schedule 25.0 data on key indicators of Household social consumption in India: health, collected by the National Sample Survey Organization (NSSO) during 2017–18. The 75th round survey was aimed at generating basic quantitative information on the health sector. The NSS has adopted multistage stratified sampling design with census villages and urban blocks as the first-stage units for the rural and urban areas, respectively, and households as the second-stage units for ensuring regional and social group representation. A detailed methodology of data collection and sampling design was published elsewhere [33]. The major objective of the survey was to determine the prevalence rate at the state and national level of general morbidity by age-group and gender, as well as of specific categories of ailment. The survey collected data from 555,372 individuals. We remove the missing cases (83 cases) from the data to provide better estimates. The sample size for this study was 555,289 individuals, for which data was used in the present study for the analysis.

### Outcome variable

A direct question was asked to the respondent regarding the nature of ailment such as particular medical treatment received as an in-patient of a medical institution during the last 365 days ‘Reported Diagnosis and/or Main Symptom’. The survey collected data about 89 diseases/symptoms of the household members. Asthma was the binary outcome variable of this study; if a person reported diagnosis of asthma it was coded as ‘1’ and ‘0’, otherwise.

### Exposure variables

The predictor variables included age of the individual (less than 5, 5–14, 15–29, 30–44, 45–65, and 65 + years), sex (male and female), marital status (never married, currently married and others), educational status (no education, below primary, primary and middle, secondary and above), religion (Hindu, Muslim, and others), caste (scheduled caste, scheduled tribe, other backward class, and others). The caste system in India has its roots in the earlier *varna* (color) system. The *varnas* represented a social hierarchy with purity and pollution-related notions, which is based on the principle that some works were considered pure and some impure or polluted. Accordingly, the system was setup to delegate the various activities to particular groups of people. Thus, the Scheduled Caste includes a group of the population that is socially and financially/economically segregated by their low status as per Hindu caste hierarchy. The Scheduled Castes (SCs) and Scheduled Tribes (STs) are among the most disadvantaged socio-economic groups in India. The OBC is is a group of intermediate categories identified as “educationally, economically and socially backward”. The “other” caste category is identified as having higher social status [34, 35]. Place of residence (rural and urban), monthly per capita consumption expenditure (MPCE) (poorest, poorer, middle, richer, and richest), cooking fuel (clean and others), source of drinking water (improved and unimproved), type of toilet facility (improved and unimproved), household size (less than 4 members and 4 or more member), and garbage disposal (have an arrangement and no arrangement).

### Statistical analysis

Descriptive statistics were used to show the distribution of the study population. Further, bivariate and multivariable analysis was used to identify the factors associated with Asthma. Moreover, wealth quintile was the key variable to measure the economic status of the household. To study the variation in asthma, health expenditure, choice of healthcare facility etc. across the population at different levels of living, a measure of the level of living was derived for each surveyed household based on information collected on its usual monthly consumer expenditure. This allowed estimates to be generated separately for 5 different equal-sized classes of the population at different quintile class of household expenditure and also known as monthly per capita consumption expenditure (MPCE) [36]. The study used household monthly per capita expenditure (Rupees) for decomposition analysis and the calculation of Concentration Index (CI), the study used MPCE which has divided into five equal sizes of the population.

### Concentration index

Concentration index represents the magnitude of inequality by measuring the area between the concentration curve and line of equality and calculated as twice the weighted covariance between the outcome and fractional rank in the wealth distribution divided by the variable mean.

The concentration index can be written as follows:

$${\varvec{C}} = \frac{2}{{\varvec{\mu}}}{\varvec{cov}}\left( {{\varvec{y}}_{{{\varvec{i}},}} {\varvec{R}}_{{\varvec{i}}} } \right)$$

where C is the concentration index; $y_{i}$ is the outcome variable index; ***R*** is the fractional rank of individual ***i ***in the distribution of socio-economic position; ${\varvec{\mu}}$ is the mean of the outcome variable of the sample and ${\varvec{cov}}$ denotes the covariance [37]. The index value lies between − 1 to + 1.

If the curve lies above the line of equality, the concentration index takes a negative value, indicating a disproportionate concentration of inequality among the poor (pro-rich). Conversely, if the curve lies below the line of equality, the concentration index takes a positive value, indicating a disproportional concentration of inequality among the rich (pro-poor). In absence of socio-economic related inequality, the concentration index is zero.

### Decomposition of the concentration index

The study used Wagstaff decomposition analysis to decompose the concentration index. Wagstaff’s decomposition demonstrated that the concentration index could be decomposed into the contributions of each factor to the income-related inequalities [38]. Based on the linear regression relationship between the outcome variable $y_{i}$, the intercept α, the relative contribution of $x_{ki}$ and the residual error $\varepsilon_{i}$

$$y_{i} = \alpha + \sum \beta_{k} x_{ki} + \varepsilon_{i}$$

where $\varepsilon_{i}$ is an error term, given the relationship between $y_{i}$ and $x_{ki}$, the CI for y (C) can be rewritten as:

$$C = \sum \left( {\frac{{\beta_{k} \overline{x}_{k} }}{\mu }} \right)C_{k} + \frac{GC\varepsilon }{\mu }/\mu$$

where $\mu$ is the mean of $y_{i}$, $\overline{x}_{k}$, is the mean of $x_{k}$, $\beta_{k}$ is the coefficient from a linear regression of outcome variable, $C_{k}$ is the concentration index for $x_{k}$ (defined analogously to C, and GC_ɛ_ is the generalized concentration index for the error term ($\varepsilon_{i}$).

Here C is the outcome of two components: First, the determinants or ‘explained’ factors. The explained factors indicate that the proportion of inequalities in the outcome (Asthma) variable is explained by the selected explanatory factors, i.e., x_k_. Second, a residual or ‘unexplained’ factor $\left( {\frac{GC\varepsilon }{\mu }/\mu } \right)$, indicating the inequality in health variable that cannot be explained by selected explanatory factors across various socioeconomic groups.

The analysis was adjusted for complex survey design (in this case multistage sampling) by using svyset command in STATA 14. The svyset command also adjusted the estimates for survey weights.

## Results

Table 1 presents the socio-economic profile of the study population in India. About 3.3% of the population belong to the age group 65 years and above. About 51.7% of the population was male and 48.3% was female. Nearly, 50.5% of the population was currently married and 44.4% was never married. Almost 26.1% of the population was not educated and 30.3% was having education secondary and above. About 8 in 10 people in India belong to the Hindu religion. About one-tenth of population was from the Scheduled Caste category and additionally, about 2 in 10 people belong to the Scheduled Caste category. About 70.5% of the population belong to a rural place of residence. Nearly 20.5% of the population belong to poorest wealth quintile and 19.9% of the population belong to richest wealth quintile. Nearly, 55.2% of household used clean cooking fuel, 96.5% used improved source for drinking water and 75.2% used improved toilet facilities. About 83% of households had a household size of four or more. Nearly, 59% of households had no arrangement for garbage disposal.

**Table 1:** Socio-economic and demographic profile of study population, 2017–18

Background characteristics	Percentage	Sample
Age (in years)		
< 5	7.3	64,720
05–14	19.2	90,907
15–29	28.0	1,53,103
30–44	22.1	1,16,038
45–65	20.3	1,09,656
65 +	3.3	20,865
Sex		
Male	51.7	2,83,193
Female	48.3	2,72,096
Marital status		
Never married	44.4	2,42,387
Currently married	50.5	2,86,022
Others^§^	5.2	26,880
Educational status		
No education^#^	26.1	1,47,250
Below primary	16.4	84,648
Primary and middle	27.3	1,41,734
Secondary & above	30.3	1,81,657
Religion		
Hindu	81.1	4,12,632
Muslim	14.1	83,047
Others^$^	4.7	59,610
Caste group		
Scheduled Tribe	9.1	75,256
Scheduled Caste	19.6	94,087
Other Backward Class	44.9	2,22,876
Others	26.4	1,63,070
Place of residence		
Rural	70.5	3,25,988
Urban	29.5	2,29,301
MPCE		
Poorest	20.5	1,12,807
Poorer	21.1	1,12,365
Middle	19.1	1,02,645
Richer	19.5	1,05,701
Richest	19.9	1,21,771
Cooking fuel		
Clean^£^	55.2	3,40,801
Others	44.8	2,14,488
Source of drinking water		
Improved^€^	96.5	5,31,692
Unimproved	3.5	23,597
Type of toilet facility		
Improved^¥^	75.2	4,62,311
Unimproved	24.8	92,978
Household size		
Less than 4 member	17.0	68,129
4 or more member	83.0	4,87,160
Garbage disposal		
Have arrangement	41.0	2,68,077
No arrangement	59.0	2,87,212
Total	100.0	5,55,289

*MPCE* Monthly per capita consumption expenditure

^§^Includes widow, separated and divorced

^*#*^No education also includes those who never attended school

^$^Includes Christianity, Sikhism, Jainism, Buddhism, Zoroastrianism, and Others

^£^Clean only includes LPG and Others includes firewood and chips, other natural gas, dung cake, kerosene, coke, coal, gobar gas, other biogas, charcoal, no cooking arrangement and others which were used for any purpose

^€^Includes bottled water, piped water in dwelling/premises/yard, piped water outside, tube-well/bore-well (inside or outside premise), protected well (inside or outside premise), protected spring/pond and community RO plant

^¥^Includes flush/pour flush latrine to: piped sew, septic tank, pit latrine

Table 2 represents the prevalence of asthma and its logistic regression estimates by background characteristics in India. Only the logistic regression estimate will be interpreted as they provide the adjusted figures. Individuals aged 65 + years had 67.92 times significantly higher likelihood to suffer from asthma in comparison to individuals less than five years [OR: 67.92; CI: 37.75–122.2]. Females had 14% significantly lower likelihood to suffer from asthma than males [OR: 0.86; CI: 0.76–0.98]. Individuals who were divorced/separated/widowed were 47% significantly higher likelihood to suffer from asthma in comparison to individuals from currently married status. Individuals with no educational status had 81% significantly higher likelihood to suffer from asthma than individuals who had secondary and above educational status [OR: 1.81; CI: 1.50–2.20]. Individuals from the Muslim religion had 29% significantly higher likelihood to suffer from asthma than individuals from Hindu religion [OR: 1.29; CI: 1.10–1.52]. Individuals from the urban place of residence had 45% significantly higher likelihood to suffer from asthma than individuals from a rural place of residence. The individual from Scheduled Tribe had 54% lower likelihood to suffer from Asthma in reference to individuals from other caste category [OR: 0.46; CI: 0.34–0.61].

**Table 2:** Prevalence of Asthma and logistic regression estimates by background characteristics, 2017–18

Background characteristics	Prevalence (per 1000)	OR [95% CI]
Age (in years)		
< 5	0.1	Ref
05–14	0.5	1.63*(0.92–2.87)
15–29	0.4	2.41***(1.39–4.18)
30–44	1.0	6.72***(3.71–12.18)
45–65	5.1	23.15***(12.95–41.37)
65 +	17.3	67.92***(37.75–122.2)
Sex		
Male	2.0	Ref
Female	2.1	0.86**(0.76–0.98)
Marital status		
Never married	0.5	1.30(0.91–1.84)
Currently married	2.5	Ref
Others^§^	10.8	1.47***(1.26–1.72)
Educational status		
No education^#^	3.5	1.81***(1.50–2.20)
Below primary	1.6	1.71***(1.36–2.15)
Primary and middle	1.8	1.41***(1.17–1.69)
Secondary and above	1.3	Ref
Religion		
Hindu	2.0	Ref
Muslim	2.0	1.29***(1.10–1.52)
Others^$^	2.5	0.79*(0.62–1)
Caste group		
Scheduled Tribe	1.4	0.46***(0.34–0.61)
Scheduled Caste	2.4	1.01(0.84–1.21)
Other Backward Class	1.8	0.93(0.81–1.07)
Others	2.4	Ref
Place of residence		
Rural	1.9	Ref
Urban	2.3	1.45***(1.24–1.69)
MPCE		
Poorest	1.5	Ref
Poorer	1.9	1.19(0.96–1.47)
Middle	2.0	1.45***(1.18–1.79)
Richer	2.0	1.46***(1.18–1.8)
Richest	2.9	1.76***(1.43–2.17)
Cooking fuel		
Clean^£^	2.0	Ref
Others	2.1	1.37***(1.19–1.59)
Source of drinking water		
Improved^€^	2.0	Ref
Unimproved	2.1	1.34**(1.03–1.75)
Type of toilet facility		
Improved^¥^	2.1	Ref
Unimproved	1.9	0.94(0.79–1.13)
Household size		
Less than 4 member	1.7	0.71***(0.62–0.82)
4 or more member	3.7	Ref
Garbage disposal		
Have arrangement	2.0	Ref
No arrangement	2.1	1.13*(0.98–1.3)
Total	2.0	

^*^if *p* < 0.01 **if *p* < 0.05 ***if *p* < 0.1; OR: Odds Ratio; CI: Confidence Interval; MPCE: Monthly per capita consumption expenditure

Includes widow, separated and divorced

^*#*^No education also includes those who never attended school

^$^Includes Christianity, Sikhism, Jainism, Buddhism, Zoroastrianism, and Others

^£^Clean only includes LPG and Others includes firewood and chips, other natural gas, dung cake, kerosene, coke, coal, gobar gas, other biogas, charcoal, no cooking arrangement and others which were used for any purpose

^€^Includes bottled water, piped water in dwelling/premises/yard, piped water outside, tube-well/bore-well (inside or outside premise), protected well (inside or outside premise), protected spring/pond and community RO plant

^¥^Includes flush/pour flush latrine to: piped sew, septic tank, pit latrine

Further, individuals from richest wealth quintile had 76% significantly higher likelihood to suffer from asthma than individuals from poorest wealth quintile [OR: 1.76; CI: 1.43–2.17]. Individuals from the household with the unclean source of cooking fuel had 37% significantly higher likelihood to suffer from asthma than individuals from the household with a clean source of cooking fuel [OR:1.37; CI: 1.19–1.59]. Individuals from the household with an unimproved source of drinking water had 34% significantly higher likelihood to suffer from asthma than individuals from the household with an improved source of drinking water [OR: 1.34; CI: 1.03–1.75]. Individuals from the household with 4 or less members had 29% significantly lower likelihood to suffer from asthma than individuals from the household with 4 or more members [OR: 0.71; CI: 0.62–0.82].

Table 3 represents the state-wise prevalence and concentration index (CCI) value for asthma in India. Daman and Diu had the highest prevalence of asthma (20.7%) followed by Kerala (8.1%) and West Bengal (4.9%). Additionally, highest value for concentration index for asthma was for Chandigarh (0.694; *p* < 0.05) followed by Mizoram (0.560; *p* < 0.5) and West Bengal (0.395; *p* < 0.05).

**Table 3:** State-wise prevalence and concentration index value for Asthma, 2017–18

States	Prevalence (per 1000)	Index value	St. error
Jammu & Kashmir	1.1	− 0.091	0.136
Himachal Pradesh	3.1	0.102	0.103
Punjab	3.6	− 0.116	0.074
Chandigarh	3.0	0.694*	0.266
Uttarakhand	1.3	− 0.140	0.176
Haryana	0.9	0.254*	0.151
Delhi	0.8	− 0.557*	0.249
Rajasthan	2.0	0.382*	0.077
Uttar Pradesh	2.1	0.029	0.051
Bihar	0.8	0.045	0.124
Sikkim	0.2	0.455	0.694
Arunachal Pradesh	0.4	− 0.048	0.312
Nagaland	0.0	–	–
Manipur	0.5	− 0.150	0.231
Mizoram	1.0	0.560*	0.208
Tripura	0.2	0.536	0.467
Meghalaya	0.0	–	–
Assam	0.8	0.076	0.154
West Bengal	4.9	0.395*	0.047
Jharkhand	1.6	0.104	0.115
Orissa	2.0	0.378*	0.093
Chhattisgarh	0.8	− 0.298*	0.163
Madhya Pradesh	1.3	0.174*	0.091
Gujarat	1.5	0.298*	0.102
Daman & Diu	20.7	0.112	0.163
Dadra & Nagar Haveli	0.0	–	–
Maharashtra	2.0	− 0.361*	0.061
Andhra Pradesh	2.9	0.075	0.082
Karnataka	0.7	− 0.004	0.142
Goa	0.3	0.792	0.706
Lakshadweep	0.4	0.966	0.871
Kerala	8.1	0.032	0.045
Tamil Nadu	1.2	0.129	0.101
Pondicherry	0.0	–	–
Andaman & Nicobar Island	0.6	0.638	0.505
Telangana	1.8	− 0.281*	0.112

*St. Error* Standard error; *if *p* < 0.05

**Fig. 2:**
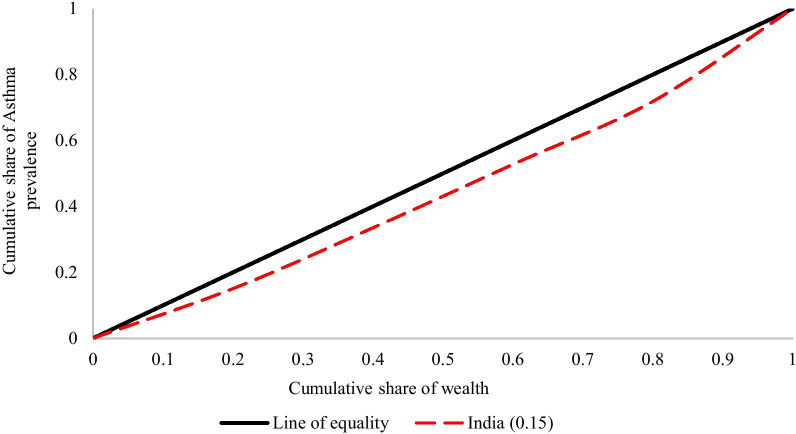
Concentration curve for Asthma prevalence among the Indian population, 2017–18

Figure 2 reveals a concentration curve for asthma prevalence among the Indian population and it was found that asthma was more concentrated among individuals from higher socioeconomic status (CCI: 0.15; *p* < 0.05). The adjusted (Erreygers normalization) CCI was 0.005.

Table 4 represents decomposition analysis estimates for asthma prevalence in India. Coefficients were obtained by applying logit regression. Absolute contribution is the product of elasticity and CCI whereas the percentage contribution is the proportion of absolute contribution multiplied by 100. In explaining socio-economic inequality for asthma, richest wealth status (53.9%) was the most significant contributor in explaining the majority of the inequality followed by the urban place of residence (37.9%) and individual from age group 45–65 years (33.3%). Additionally, individual aged 65 years and above (27.9%) and household size less than four members (14.7%) contributed in explaining socio-economic inequality for asthma.

**Table 4:** Decomposition analysis estimates for asthma prevalence among Indian population, 2017–18

Background characteristics	Coefficients	Elasticity	CI	Absolute contribution	%contribution	Total contribution
Age (in years)						
< 5						
05–14	0.49	0.000	− 0.115	0.000	− 4.7	61.0
15–29	0.88	0.000	0.012	0.000	1.0	
30–44	1.91	0.000	0.028	0.000	3.5	
45–65	3.14	0.001	0.081	0.000	33.3	
65 +	4.22	0.001	0.136	0.000	27.9	
Sex						
Male						
Female	− 0.15	0.000	− 0.001	0.000	0.0	0.0
Marital status						
Never married	0.26	0.000	− 0.047	0.000	− 3.9	1.6
Currently married						
Others^§^	0.39	0.000	0.067	0.000	5.5	
Educational status						
No education^#^	0.60	0.000	− 0.169	0.000	− 13.9	− 19.9
Below primary	0.54	0.000	− 0.095	0.000	− 3.9	
Primary and middle	0.34	0.000	− 0.051	0.000	− 2.1	
Secondary above						
Religion						
Hindu						
Muslim	0.25	0.000	− 0.035	0.000	− 1.4	− 1.4
Others^$^	− 0.24	0.000	0.291	0.000	0.0	
Caste group						
Scheduled Tribe	− 0.79	0.000	− 0.301	0.000	0.0	− 5.2
Scheduled Caste	0.01	0.000	− 0.149	0.000	− 6.1	
Other Backward Class	− 0.07	0.000	− 0.021	0.000	0.9	
Others						
Place of residence						
Rural						
Urban	0.37	0.000	0.462	0.000	37.9	37.9
MPCE						
Poorest						
Poorer	0.17	0.000	− 0.257	0.000	− 10.6	57.4
Middle	0.37	0.000	0.035	0.000	1.4	
Richer	0.38	0.000	0.309	0.000	12.7	
Richest	0.57	0.000	0.657	0.000	53.9	
Cooking fuel						
Clean^£^						
Others	0.32	0.000	− 0.277	0.000	− 22.7	− 22.7
Source of drinking water						
Improved^€^						
Unimproved	0.29	0.000	− 0.038	0.000	0.0	0.0
Type of toilet facility						
Improved^¥^						0.0
Unimproved	− 0.06	0.000	− 0.361	0.000	0.0	
Household size						
Less than 4 member	− 0.34	− 0.001	− 0.060	0.000	14.7	14.7
4 or More member						
Garbage disposal						
Have arrangement						
No arrangement	0.12	0.000	− 0.190	0.000	− 23.4	− 23.4
Actual CI				0.0002		
Calculated CI				0.1500		
Residual				0.1497		

*MPCE* Monthly per capita consumption expenditure; *CI* Concentration Index; *%* percentage

^§^Includes widow, separated and divorced

^*#*^No education also includes those who never attended school

^$^Includes Christianity, Sikhism, Jainism, Buddhism, Zoroastrianism, and Others

^£^Clean only includes LPG and Others includes firewood and chips, other natural gas, dung cake, kerosene, coke, coal, gobar gas, other biogas, charcoal, no cooking arrangement and others which were used for any purpose

^€^Includes bottled water, piped water in dwelling/premises/yard, piped water outside, tube-well/bore-well (inside or outside premise), protected well (inside or outside premise), protected spring/pond and community RO plant

^¥^Includes flush/pour flush latrine to: piped sew, septic tank, pit latrine

## Discussion

There is a common notion that some disparities persist in terms of health outcomes across the poor and better-off section of society. And the problem intensifies when individuals are left undiagnosed due to lack of awareness and access to health care services. Ample evidence revealed that changing lifestyles and growing level of stress in day-to-day life easily triggers asthma in the richer section of society [39–41]. Thus, the study explored the factors associated and socio-economic inequality in the asthma prevalence and the contribution of various factors in those inequalities in India. Additionally, we have shown significant differences in state-wise prevalence rates of asthma in India.

The study reported that females had lower asthma prevalence as compared to males. In general, childhood asthma prevalence is higher in boys than in girls (especially before puberty). However, asthma prevalence becomes more prevalent in female than in males in adulthood. The inconsistent finding of our study can be a result of not stratifying by age groups while analysing the gender factor. We have also highlighted an unexpected pattern of higher prevalence of asthma among individuals with higher economic status measured by MPCE. The finding is contrary to many Western studies that showed that poor economic status and low income as risk factors for the development of asthma, asthmatic wheeze and chronic productive cough [42–44]. It is revealed that the higher prevalence of asthma found in poor compared to the affluent population in developed nations and in affluent compared to poor population in developing nations, reflects cultural and contextual differences [45]. The increased access to healthcare among people with higher economic status may explain the current finding where improved healthcare system contributes to the ascertainment of diseases such as asthma among the economically better-off populations. Studies also reported the higher likelihood of under-diagnosis and under-reporting of non-communicable diseases including asthma among lower socioeconomic groups in India [32].

Consistent with previous studies [43, 46], the contribution of educational status in the socioeconomic inequality in asthma prevalence was higher than any other socioeconomic variables in the study. The results are also in accordance with several studies that found the low educational level to be strongly associated with asthma and respiratory symptoms [42, 44, 47]. Importantly, the positive association of wealth quintile with asthma prevalence and simultaneously the negative association of education with asthma in the current analysis suggest future investigation of underlying mechanisms in such associations. Further, the finding that higher the size of household greater the prevalence of asthma is inconsistent with previous studies that reported the inverse association of the number of siblings with the prevalence of asthma and called it as ‘sibling effect’ [48, 49]. Similarly, odds of suffering from asthma was higher among men than women in our study which is contrary to several earlier studies in India that revealed a higher prevalence of the disease among women who are more exposed to poor housing conditions [50, 51]. Besides, urban rates of asthma prevalence were higher than rural rates in our study which confirms the finding that urbanization by which exposure to biomass fuel smoke increases is an environmental risk factor of asthma [52].

In a country where large proportion of the population still relies on solid and biomass fuels for cooking, a significant association was found between cooking fuel and the prevalence of asthma disease in the present study. It is found that clean cooking fuel is a protective factor against asthma which is consistent with earlier studies [51, 53, 54]. Further, an increased risk of asthma was found among the people from households that have no garbage disposal arrangements. Another study that evaluated the prevalence of asthma in relation to a residence in houses built on a former dumping area containing industrial and household wastes has shown similar finding that the risk of asthma was higher in the dump cohort than people living outside the site [55]. Consistent with a recent study in India [26], a significant association of improved source of drinking water with lower asthma prevalence was also found in the present study which is supported by evidence that shows exposure to heavy metals and arsenic in drinking water increase the prevalence of respiratory illnesses [56–58]. Other potential mechanisms for such associations including the exposure to allergens need to be further explored in future studies.

The highest prevalence of asthma in the coastal states of Kerala, and West Bengal and the UT of Daman& Diu may be attributed to its geographical features, where people consume more fish that may contribute to the higher burden of asthma in these States/UT [51]. We also found a relative difference between the lowest and highest region-wise prevalence rates that ranged between 0 to 20.7 individuals suffering from asthma per thousand individuals indicating that there are wide regional variations in the prevalence of asthma in India. Although the similar findings are shown in previous studies [26, 51], the reasons for the variations are unclear and require further investigation.

Large nationally representative sample is the strength of our study, which allows comparisons between states and urban–rural settings, and the ability to examine socio- economic and housing patterns of asthma risk. However, the study is limited by its cross-sectional design. Additionally, biological or social factors related to asthma were not measured in this study which may have influenced and contributed to the gender and place of residence-related differences observed. Besides, the higher prevalence of asthma in older age groups in comparison to younger population might be due to potential bias in reporting the disease such as potential misclassification between chronic obstructive pulmonary disease (COPD) and asthma in the older age groups.

## Conclusion

Advancing age, male sex, residence in the urban area, lower education, higher MPCE and poor housing conditions such as unclean cooking fuel, unimproved source of drinking water and unarranged garbage disposal were associated with significantly higher odds of having asthma. Due to the heterogeneous nature of asthma, associations between different SES indicators and asthma can be complex and may point in different directions. Hence, considering the concentration of asthma prevalence in vulnerable populations and its long-term effect on general health, a comprehensive programme to tackle chronic respiratory diseases and asthma, in particular, is urgently needed.

Future studies are warranted on the higher prevalence of asthma among wealthy people observed in the current study. Besides, a state-specific analysis must be conducted to explore the substantial differences in asthma prevalence and different socioeconomic and environmental risk factors in Indian states. And further longitudinal studies should be conducted to confirm the temporal sequence of the results and further elucidate the impact of socioeconomic and contextual disadvantages on the incidence of asthma over the course of time.

## Data Availability

The study utilises secondary source of data which is freely available in public domain through http://mospi.nic.in/NSSOa.
